# The Income Tax

**Published:** 1914-01

**Authors:** 


**A Monthly Review of Medicine and Surgery**

EDITOR AND PUBLISHER DR. A. L. BENEDICT, 228 Summer St. corner of Elmwood Ave., Buffalo. (Address for all communications. Please make personal and telephone calls before 1 P. M.)

Third, in age, in the western continent. Independent but supports professional organization. News limited mainly to western N. Y. and Penn.

Subscription $2.00 a year; $3.00 for two years in advance. Changes and errors in address or failure or duplication of delivery, should be reported immediately.

YEARLY VOLUME 69 JANUARY, 1914 No. 6

### The Income Tax.

We feel that a discussion of this tax is allowable in a medical journal, both as a matter of public interest and because it applies directly to some physicians though not to as large a proportion of the profession as might be desired. We have, indeed, been asked specifically for advice on the matter by several acquaintances.

We do not share the antagonism which many have expressed regarding this tax. Indirect taxation is popular because it is not felt, or when felt it strikes individually, perhaps hard and unjustly, but the individual complaint is lost on the ears of the general public. We believe that every tax should be felt and that it should bear as evenly as possible on the public. For instance, when a man buys a box of cigars in Holland for two cents apiece, pays a duty of five cents apiece and then fifteen cents for a revenue stamp for the box, he may rightly feel that the Government has been extortionate, that there is no particular reason for protecting an industry that is, on the whole, undesirable, so that it can maintain a schedule of prices that is extortionate as compared with most other countries. So, too, the inheritance tax rather tends to disfavor the distribution of wealth, and by imposing an extra tax on real estate which is already constantly taxed by city, county and state to the amount of 20 to 30 per cent. of its income, it simply takes advantage of a transient change of title. The income tax is ideal in that it applies to actual available wealth, that it does not tend to confiscate nominal capital of no actual value, and that it does not discriminate between different means of earning a living or accumulating wealth.

The income tax is also conservatively and properly socialistic in that it increases the burden of taxation by a series of steps so as to place the burden progressively on those best able to bear it.

Against the income tax may be urged the facts that it encourages a system of governmental espionage, that it involves the increase of the horde of office holders already too numerous and expensive, that these items involve the possibility of blackmail and graft of one kind or another. One of the best ways to become prosperous is to quit spending money, and we believe that, with reasonable allowance for expenditures that will save loss or produce an income, this method may be followed by governments as well as individuals. Those who dislike warfare have made suggestions along this line. Without committing ourselves to such means of economy, we may express the personal willingness to run the chances of future catastrophy by economizing in military and naval matters and following a national policy of minding our own business.

The income tax is not a perfected measure and it will undoubtedly result in much litigation to ascertain the meaning of some of its provisions, as applied to individual cases. But pessimists assert that this is one of the prime functions of all legislation, since legislators are mostly lawyers. With this cynicism we can scarcely agree. The general policy of American government, at least of the national government, has been one of fairness and reasonableness, even of leniency in regard to technical violations of law.

It seems to us that no one can justly complain that the lower limit of liability to the income tax has been placed at $3,000 for a single person, nor of the amount (1% on incomes above this exemption), nor of the increase of percentage after an income of $20,000 is reached. But in view of the long continued agitation for more universal family life, for the encouragement of the rearing of children, etc., actual experience with the law is scarcely necessary to show that the increased exemption of $4,000 for a married couple is subject to criticism. A single man with a net income of $3,000 is much better off, financially, than a married man with an income of $4,000. We believe, too, that any man who is supporting a near relative—the reason for this limitation is apparent on reflection—whose sex or age warrants exclusion from gainful occupation, should have the same exemption as one supporting a wife. It is more than a corollary to this proposition, to say that exemption should be allowed for children, not only because they involve actual expense and greater responsibility, but also because every child well brought up is an asset to the country. While it is not likely that the comparatively small amount of the tax will actually discourage either marriage or child rearing—this would be choking on a gnat after swallowing a camel—it would be well in these days in which bachelor taxes are seriously considered, to apply the same principle negatively.

Since definite criticisms of the law have, in a sense, been called for, we would suggest that on the relative basis of exemption of $3,000 for a single man there should be an exemption of $5,000 for a married man, an exemption of $1,000 for any near relative over age actually supported, but providing that for males over 21 the exemption should be shown to be warranted either on the ground that support was being given to a student or to one incapacitated, mentally or physically, as by senility; and that for minor children a per capita exemption of $500 should be allowed.

While the additional exemption of a married man is correct on the principle of support of a housewife or social companion, it seems to us that a woman actually owning property—not merely turned over by her husband to avoid responsibility or for insurance—or actually engaged in a gainful occupation, either independently of or in partnership with or as an employee of her husband, should have the same exemption as a single woman. This principle applies to a good many of the women of our profession. There is no obvious reason why a tax should be levied simply because a woman is married or why a happy marriage should be taxed and an unhappy one untaxed. One of our patients left with the care of three small children by the death of his wife has pointed out a further injustice in taxing widowers as single men. He not only has the natural expenses and responsibilities of a married man, but both of these are actually greater than if his wife were alive.

### POINTS OF SPECIAL INTEREST TO PHYSICIANS.

It should be distinctly understood that the exempt income on which a tax is not to be paid is $3,000 for single men or women, $4,000 for a married couple, with or without children. On all incomes above these limits 1 per cent. is charged, up to $20,000. If any of our readers have incomes beyond this limit they can look up the further provisions. It should be understood that the 1 per cent. tax is paid on net incomes and only on the part of the net income that exceeds the exemption. A similar tax applies to corporations of various kinds, but such partnerships and corporations as physicians are liable to be directly interested in are, we understand, separated into individuals for purposes of taxation. Such a partnership or corporation must not, however, attempt to evade the law by fictitious expenditures or by holding back undivided surplus beyond a reasonable amount necessary to meet current expenses.

Returns must be made to the local Internal Revenue Office between January 1 and March 1 on blanks furnished, and, for the present year, incomes, exemptions and tax are calculated from March 1, so that the pro rata exemptions would be, respectively, $2,500 and $3,333.33. Contrary to the original announcements, returns need not be made by anyone whose income falls short of these amounts.

The law contains various provisions for the bona fide exclusion of bad debts. Physicians have generally followed the plan of putting down their earnings as actually received. In this way a bad debt never appears as a nominal asset on the cash account and, as many accounts are carried over from one or several years previously while many current earnings will not be collected till the next or following years, an ultimate adjustment takes place automatically. We are informed by the local office that the report of actual receipts, as paid, in any one year, will undoubtedly be accepted as a fair basis for taxation. It will doubtless happen that many physicians have incomes so near the limit that they will pay taxes intermittently. While there would be little temptation to postpone the collection of an account for the sake of the 1 per cent. involved, and while such a course might disturb an average so as to increase the tax for a given year, any connivance to adjust incomes in this way would probably be severely punished. In fact, irrespective of the ethics of the matter, any attempt to evade the tax is subject to heavy fine and even imprisonment.

The matter of "withholding at the source" seems to us a very undesirable feature of the law, as vesting private individuals and firms with governmental rights and imposing on them a responsibility which they are no more likely to discharge than the individual ultimately taxed. We understand, however, that this does not apply to rentals, mortgage interest and other returns on invested capital except when the individual item is of large amount or, as in the case of certain coupons, when it is a part of a taxable investment. As a rule, we believe this clause will not apply to physicians' investments. If it does, a deduction can be claimed in paying the remainder of the tax or a rebate if the total income does not exceed the limit of exemption. Probably, in many cases, small bond holders will sacrifice the small amount of the tax rather than go to this trouble.

It should be definitely understood that the word income refers to net receipts, business expenses being deducted from the gross income. Office rental, wages to professional assistants, telephone rates, drugs and instruments, the expenses of an automobile maintained for business use, dues in medical societies, traveling expenses in visiting patients and probably in attending medical meetings, etc., are properly included as business expenses. We have seen a statement that the initial expense of an automobile will not be allowed. This is unjust if true, as an automobile is not a permanent improvement in the sense of an addition to a factory. However, practically, this ruling, if supported, would work little hardship, as depreciation could legitimately be charged, year by year, so that the ultimate result would be about the same. It has been questioned whether a physician is justified in counting part of his house rental, wages of domestic servants, etc., as professional expense. The fact that physicians who have offices in their residences usually pay a higher gross rental and more wages for domestic help than others of the same circumstances justifies the contention that he should deduct a reasonable sum covering the upkeep of his office, such as he would have to pay for room and attendance if he rented of someone else.

Any person owning his home must include the net value of the use of it as part of his income. Whether he should rate it proportionate to rentals of similar property and then deduct taxes, interest and repairs or whether he should calculate the interest on the investment and make the same deductions, is a debatable point, especially when, as in many cities, it is usually much cheaper to rent than to own a home, if one counts interest. We venture to assert that, in the majority of instances, if one calculates that he should receive 6 per cent. on the capital invested in a home, and deducts from this taxes, repairs and a fair allowance for office rental, there will be practically no balance left.

The taxable income includes both earnings—or, rather, collections—and return from investments. Gifts and legacies are not taxable, but the income which they yield as capital is taxable. Physicians holding real estate may, of course, deduct taxes, interest and ordinary repairs in estimating the net income from such a source. Money paid for building or for making extensive alterations calculated to increase the earning value of the investment is not counted as an expense but as shifting of capital. In addition to the regular expense of real estate, a fair amount may be charged against the gross returns for depreciation. Except in the case of mines, no definite limit is given. For ordinary buildings we should estimate that 3 per cent. of cost of construction per annum would represent a fair allowance, but this is a personal estimate, without authority, and based on the assumption that the average building will deteriorate so as to require reconstruction in about 33 years. Loss by fire, storm, etc., not covered by insurance is also provided for. For minor losses the repairs necessary will provide for this estimate automatically.

In ordinary businesses, profit and loss are important items. In medical practice, assuming that one is careful to carry insurance of various kinds, it is obvious that they do not enter into the consideration except as they affect business expenses and curtailment of income. We imagine that when a physician awakes to the fact that he has invested in worthless stock he may properly claim exemption of the actual loss. Ordinary, conservative investments do not usually offer opportunities for profit or loss except as their earning capacity is increased or diminished, and this will show automatically in the gross income. While attending the meeting of the American Gastro-enterologic Association, last spring, we heard a debate in the House as to the propriety of taxing as profit, the difference between the cost and selling price of real estate. One of the speakers contended that it was unfair to consider for purposes of taxation the gradual accumulation in value throughout a series of years, but that only the yearly profit or a profit made in direct speculation should be so considered. No definite provision has been made regarding this point, and it should be remembered that the dollars for which one sells now, represent only about 60 cents of the dollars with which a house was bought or built, say twenty-five years ago. The local revenue office confirms our opinion that loss of interest, etc., may fairly be counted in estimating the apparent profit on real estate. For instance, to take a concrete case, property bought for $6,000 twenty-five years ago and sold this year for $10,000 does not represent a profit even of $4,000 divided by 25 but, in the majority of cases, an actual loss as compared with the results of an investment in a safe mortgage. On the other hand, if a physician deals in real estate as a builder or speculator and makes prompt sales at a profit or loss, he is on the same basis as one devoting his time entirely to such business.

In some ways the medical journals of the country as well as organizations of physicians can undoubtedly assist in clearing up the tangles of the income tax law. We anticipate a genuine surprise and a questioning of good faith on account of the comparatively small number of physicians who will be liable to taxation. Many physicians who have been accustomed to rate themselves in terms of book accounts or even of gross revenue will, themselves, be surprised at the shrinkage when reduced to net terms. We are inclined to believe that the average practitioner, who requires a vehicle for his calls, spends 50 per cent. of his gross collections in strictly business expenses and that his gross collections do not usually surpass 75 per cent. of his real earnings. Another surprise is the fluctuation of professional earnings. Within the last year several successful practitioners of twenty years' experience or more have stated that their earnings have fallen off 25 per cent. or thereabouts. The ultimate loss from even brief sicknesses and vacations is far greater among physicians than among business men. In fact, we have been impressed with the frequence and success of absent treatment as applied to business by a great many. Another curious paradox of medical practice—though not without analogy in some lines of business—is that, at a certain point of growth, the necessity of increasing equipment, material and human, increases expenses disproportionately so that an actual diminution of net income may result.

